# Diabetestechnologie bei Kindern und Jugendlichen mit Diabetes mellitus Typ 1

**DOI:** 10.1007/s11428-022-00975-5

**Published:** 2022-11-08

**Authors:** Birgit Rami-Merhar

**Affiliations:** Klinische Abteilung für Pädiatrische Pulmologie, Allergologie und Endokrinologie, Universitätsklinik für Kinder- und Jugendheilkunde, Medizinische Universität Wien, Währinger Gürtel 18–20, 1090 Wien, Österreich

**Keywords:** Insulintherapie, Insulininfusionssysteme, Kontinuierliche subkutane Glukosemessung, AID (automatische Insulindosierung), Künstliche Bauchspeicheldrüse, Insulin therapy, Insulin infusion systems, Continuous subcutaneous glucose measurement, AID automated insulin-dosing system, Artificial pancreas

## Abstract

Die Behandlung des Diabetes mellitus Typ 1 (T1D) im Kindes- und Jugendalter ist komplex und stellt eine Herausforderung für die betroffenen Kinder und Jugendlichen, deren Familien und das ganze Umfeld (Schule/Kindergarten) dar. Das Ziel der Diabetestherapie besteht darin, eine möglichst normoglykämische Blutzuckerkontrolle zu erreichen, um akuten und chronischen Komplikationen vorzubeugen. Laut Registerstudien können die metabolischen Therapieziele derzeit noch nicht erreicht werden, weswegen ein Risiko für Akut- und Spätkomplikationen besteht. Weitere Therapieziele sind eine normale Entwicklung, Inklusion, Flexibilität im Alltag sowie eine hohe Lebensqualität. Abgesehen von neueren Insulinanaloga gingen auch die Entwicklungen in der Diabetestechnologie in den letzten Jahren mit großen Veränderungen und Verbesserungen in der Behandlung und Lebensqualität der betroffenen Familien einher. Die Insulinpumpentherapie, die kontinuierliche Glukosemessung sowie die automatische Insulindosierung (AID) führten zu einer signifikanten Verbesserung der metabolischen Einstellung sowie einer Reduktion der schweren Hypoglykämien und Ketoazidosen. Die Diabetestechnologie entwickelt sich ständig weiter und erfordert eine umfassende Schulung und Fortbildung der betroffenen Familien, der Betreuungseinrichtungen sowie auch des multidisziplinären Behandlungsteams. Ziel sind eine Reduktion der glykämischen Variabilität und damit ein besseres Langzeitoutcome der jungen Menschen mit T1D. Die AID ist zunehmend die Therapie der Wahl bei Kindern und Jugendlichen mit T1D. Mit weiteren Innovationen im Bereich der Diabetestechnologie ist in naher Zukunft zu rechnen.

## Abstract (en)

Treatment of type 1 diabetes mellitus (T1D) in children and adolescents is complex and a challenge for the children and adolescents themselves, their families, and their whole environment (schools/kindergartens). The aim of the diabetes therapy is to achieve near-normoglycemic glucose values and to prevent acute and chronic complications. Further aims are normal development, inclusion, flexibility in everyday life, and a high quality of live. Apart from newer insulin analogues, diabetes technology has changed and improved diabetes therapy and the quality of life of affected families. The usage of insulin pumps—continuous insulin infusion, continuous subcutaneous glucose monitoring, and automated insulin dosing (AID) has led to significant improvement in metabolic control, a reduction of severe hypoglycemia and diabetic ketoacidosis. Diabetes technology is constantly evolving and necessitates comprehensive continuing education of the families, the school/kindergarten teams, as well as the multidisciplinary team of healthcare professionals. The intention is a reduction of glycemic variability and a better long-term outcome in young people with T1D. AID is increasingly becoming the therapy of choice in children and adolescents with T1D. Further innovations in diabetes technology can be expected for the near future.

Die sehr variabel und flexibel gestaltbare Insulinabgabe über Insulinpumpe (Continuous Subcutaneous Insulin Infusion, CSII) gilt im Kindes- und Jugendalter als Therapie der Wahl. Für die kontinuierliche Glukosemessung (CGM) werden immer genauere, werkskalibrierte Systeme mit Zulassung für Therapieentscheidungen ohne zusätzlich notwendige kapillare Messung entwickelt, und sie ist im Kinder- und Jugendalter weit verbreitet. Closed-Loop-Systeme (AID [automatische Insulindosierung]) sind inzwischen auch für ein Alter <7 Jahren verfügbar und stellen zunehmend die Therapie der Wahl für Kinder und Jugendliche mit Typ‑1-Diabetes (T1D) dar.

## Einleitung

Diabetes mellitus Typ 1 ist eine der häufigsten chronischen Stoffwechselerkrankungen im Kindes- und Jugendalter, mit steigenden Inzidenzzahlen, sowohl weltweit als auch in Österreich [1, 2]. Das Ziel der Diabetestherapie besteht darin, eine möglichst normoglykämische Blutzuckerkontrolle zu erreichen, um akuten und chronischen Komplikationen vorzubeugen. Weitere Therapieziele sind eine normale Entwicklung, Inklusion, Flexibilität im Alltag sowie eine hohe Lebensqualität.

Neben der Entwicklung synthetisch hergestellter Insulinanaloga mit vorteilhafteren Wirkprofilen wurden die Therapie und das Management des T1D in den letzten beiden Jahrzehnten v. a. durch Innovationen und neuartige Behandlungsmodalitäten im Bereich der Diabetestechnologien geprägt, sowohl was die Insulinabgabe via Insulinpumpen als auch das Glukosemonitoring mithilfe von Systemen zur kontinuierlichen Glukosemessung (CGM-Systeme [CGM: „continuous glucose monitoring“]) betrifft. Diese werden als *proximale* Diabetestechnologien bezeichnet.

Zunehmend verwendete *distale* Technologien (inklusive mobiler Gesundheits-Apps, sozialer Plattformen und Patientenportale, spielbasierter Anwendungen und Tele-Health-Lösungen) werden hier aus Platzgründen nicht berücksichtigt.

## Besondere Anforderungen und Bedürfnisse

Das Management von T1D bei Kindern und Jugendlichen stellt eine besondere Herausforderung für die Personen mit Diabetes (PmD), ihre Familien und die sie betreuenden Diabetesteams dar. Bei Kleinkindern kommt es durch die Unvorhersehbarkeit des Essverhaltens und der körperlichen Aktivität bei gleichzeitig deutlichen zirkadianen Veränderungen der Insulinsensitivität zu schnellen Schwankungen der Glukosespiegel. Zusätzlich sind in dieser Altersgruppe oft sehr kleine Dosierungsschritte notwendig, möglichst wenige Injektionen sind erstrebenswert. Bei Jugendlichen am anderen Ende des pädiatrischen Altersspektrums gibt es, abgesehen von der psychosozial oft ohnehin sehr turbulenten Lebensperiode, wachstums- bzw. pubertätsassoziierte endokrine Veränderungen, die u. a. zu einem ausgeprägten Anstieg des Blutzuckersiegels in den frühen Morgenstunden führen können (Dawn-Phänomen).

## Status quo bei Kindern und Jugendlichen mit Typ‑1-Diabetes

**Figure 1:**
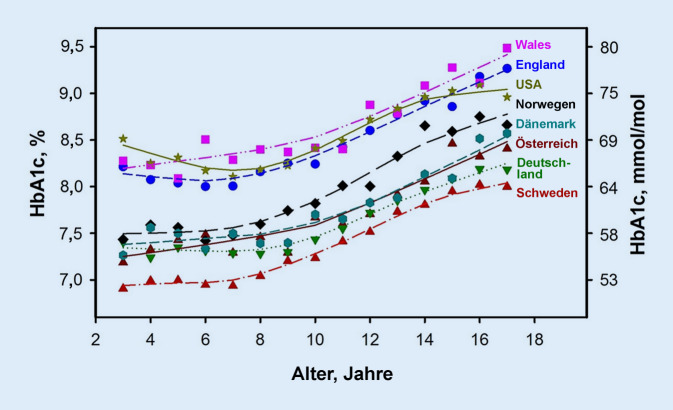

Große internationale Registervergleiche zeigen große regionale Unterschiede in der metabolischen Einstellung. Allen gemeinsam ist, dass so gut wie nie das therapeutische Ziel eines HbA_1c_ (Glykohämoglobin) < 7,0 rel% (53,1 mmol/mol) erreicht wird und dass in allen Registern in der Pubertät eine Verschlechterung zu beobachten ist ([3, 4]; Abb. 1). Die Raten für schwere Hypoglykämien und Ketoazidosen sind weiterhin zu hoch [5, 6]. Alarmierend sind auch wissenschaftliche Daten, laut welchen eine frühe Diabetesmanifestation mit einer signifikant höheren Mortalität und Morbidität (v. a. kardiovaskuläre Ereignisse) assoziiert ist und Mädchen/Frauen im Vergleich zu Knaben/Männern ein noch höheres Risiko haben [7]. Es daher notwendig, die metabolische Einstellung und das therapeutische Outcome dieser Kinder und Jugendlichen zu verbessern.

## Diabetestechnologie

### Insulinpumpentherapie

**Figure 2:**
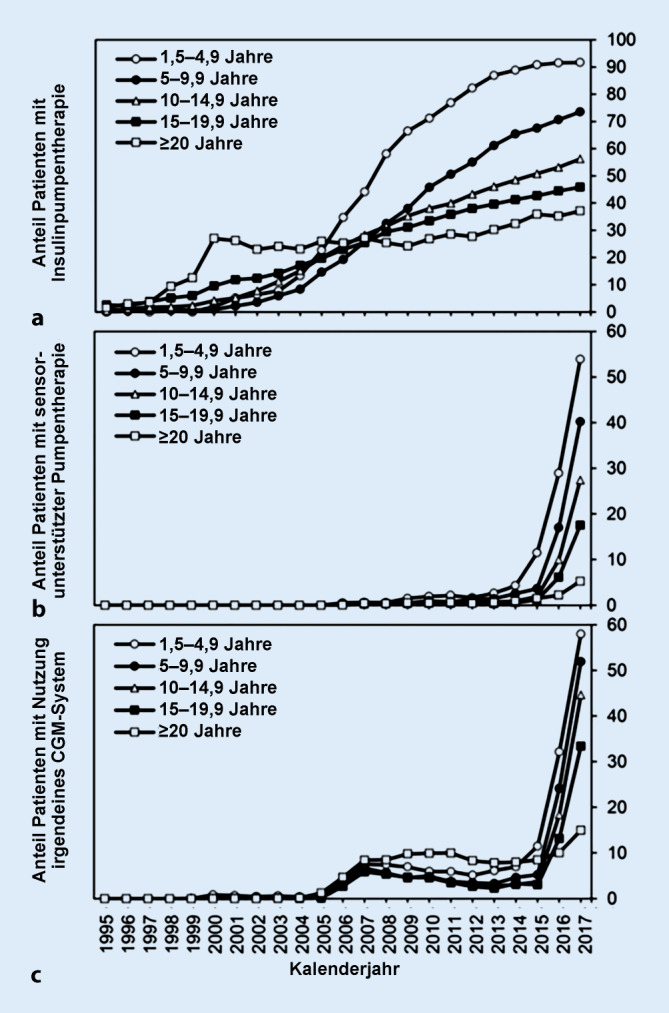

Die Insulinpumpentherapie erfüllt diese komplexen Anforderungen an das Diabetesmanagement im Kinder- und Jugendalter aufgrund der sehr variablen und subtilen Möglichkeiten der Insulindosisadaptierung aktuell am besten. Das zeigt sich sowohl in Metaanalysen prospektiver randomisierter Studien [8, 9] als auch in Beobachtungsstudien [10, 11], in denen bei Kindern und Jugendlichen mit T1D Vorteile der Pumpentherapie im Vergleich zur Basis-Bolus-Therapie mit Insulinpens im Hinblick auf verbesserte Stoffwechseleinstellung, Reduktion der Häufigkeit diabetischer Ketoazidosen und Reduktion schwerer Hypoglykämien festgestellt wurden. Die Insulinpumpentherapie gilt daher in vielen Ländern als Therapie der Wahl für Kinder und Jugendliche mit T1D und erfreut sich zunehmender Beliebtheit. In Deutschland und Österreich stieg der Anteil aller PmD mit Insulinpumpentherapie von 1 % im Jahr 1995 auf 53 % im Jahr 2017, wobei die höchsten Raten bei den jüngsten PmD zu verzeichnen sind (Abb. 2; [12]).

### Kontinuierliche Glukosemonitoringsysteme (CGM-Systeme)

Neben der Insulinabgabe über Pens oder Pumpen ist die regelmäßige Überprüfung des Blutzuckerspiegels essenzieller Bestandteil des Diabetesmanagements. Bis vor kurzem war die kapillare Messung die am weitesten verbreitete Methode des Glukosemonitorings. Idealerweise soll die Messfrequenz bei Kindern und Jugendlichen mit T1D zwischen 6 und 10 Messungen/Tag betragen [13]. Die Entwicklung und ständige Verbesserung von Systemen zum kontinuierlichen Glukosemonitoring leiteten hier wohl in den letzten Jahren einen Paradigmenwechsel ein. Die Messwerte werden bei sog. „real time“ CGM-Systemen (rt-CGM) entweder in Echtzeit (z. B. DexCom G6/G7, San Diego, CA, USA, Medtronic Guardian, Dublin, Irland) oder nur auf Abruf („on demand“) beim aktiven Scannen (Systeme zum intermittierenden Scannen eines CGM-Sensors, iscCGM [isc: „intermittent scanning“] oder Flash-CGM: FreeStyle-Libre-Systeme der Firma Abbott, Chicago, IL, USA) auf entsprechenden Lesegeräten angezeigt (eigenständige Geräte oder in Insulinpumpen oder Mobiltelefone integriert). Üblicherweise haben CGM-Sensoren eine Lebensdauer von 7–14 Tagen und werden vom Benutzer selbst ins subkutane Fettgewebe gesetzt. Es gibt aber auch einen CGM-Sensor mit einer Lebensdauer von bis zu 6 Monaten (Eversense, Roche, Basel, Schweiz), er muss s.c. implantiert werden und ist erst ab 18 Jahren zugelassen. Dieser ist in Österreich nicht mehr erhältlich.

Die Messgenauigkeit der Systeme verbesserte sich laufend, aber im hypo- und hyperglykämischen Bereich gibt es noch zumeist größere Abweichungen zu kapillaren Messungen. Der Medtronic Guardian 3 muss noch 2‑ bis 4‑mal täglich kalibriert werden, beim Guardian 4 sind keine Kalibrierungen mehr notwendig. Letzterer ist ebenso wie DexCom G6/G7 sowie FreeStyle Libre für Therapieentscheidungen zugelassen. Das bedeutet, bei solchen Systemen ist die Notwendigkeit regelmäßiger kapillarer Messungen nicht mehr gegeben, was v. a. bei Kindern zur Entlastung führt und auch bei Teenagern mit mangelnder Therapieadhärenz Vorteile bietet. Bestätigende Kapillarglukosemessungen sind dennoch bei Werten im hypo- bzw. stark hyperglykämischen Bereich empfohlen oder wenn die klinischen Symptome nicht mit den Sensorwerten übereinstimmen.

Initiale Studien und Metaanalysen zum Nutzen von CGM-Systemen waren eher zurückhaltend bzw. widersprüchlich, was den Vorteil v. a. für Kinder und Jugendliche mit T1D betrifft [14, 15]. Daten von in den letzten 10 Jahren veröffentlichten Studien unter Verwendung ständig verbesserter Sensortechnologien zeigten jedoch konsistenter, dass die Verwendung von CGM mit einer Verbesserung der HbA_1c_-Spiegel, einer Reduktion leichter bis mittelschwerer Hypoglykämien und einer Verminderung der Glukosevariabilität verbunden ist [16, 17].Sprachen erste Analysen und Richtlinien v. a. für die kombinierte Verwendung von CGM und Pumpentherapie, unterstützten jüngste Studienergebnisse die Verwendung von CGM auch als Teil der Basis-Bolus-Therapie mit Insulinpens [18, 19].

Die Nutzung von CGM-Systemen geht mit einer Verbesserung der glykämischen Einstellung einher

Die rt-CGM-Systeme verfügen über eine Reihe individuell einstellbarer Alarmfunktionen wie Hoch- und Tiefalarme bei Verlassen des Zielbereichs oder Alarme bei Glukosetrendänderungen. Die CGM-Daten mancher Sensoren können auch kontinuierlich in eine Cloud gesendet werden, auf die – wenn aktiviert – Dritte wie Eltern oder Partner über mobile Apps (z. B. DexCom G6 Follow App, LibreLink App), oder spezielle Software (Nightscout) zugreifen und auch Alarme erhalten können. Diese Möglichkeit des Remote-Monitorings (s. unten) wird v. a. bei Eltern von Klein- bis Volksschulkindern sehr geschätzt und erleichtert die Kontrolle der Glukosespiegelverläufe und die Unterstützung ihrer Kinder und betreuender Personen beim Diabetesmanagement in Kindergarten und Schule.

Aufgrund dieser rasanten technischen Entwicklung der letzten Jahre bei gleichzeitig weitestgehender Kostenübernahme durch Krankenkassen, v. a. im pädiatrischen Bereich, hat sich der Anteil an CGM-Nutzern in Deutschland und Österreich in den letzten 10 Jahren mehr als verzehnfacht [12]. Lag der Prozentsatz der PmD, die CGM verwenden, im Jahr 2006 noch bei 3 %, so waren es im Jahr 2017 bereits 38 %, die höchsten Raten findet man bei den jüngsten PmD (Abb. 2; [12]).

Bei immer verbreiteterer Anwendung von CGM-Systemen ergeben sich auch Möglichkeiten, den Erfolg der Diabetestherapie nicht nur mittels regelmäßiger Bestimmung des HbA_1c_ zu überwachen, sondern auch CGM-basierte Parameter heranzuziehen. Vielfach wird gemäß rezenter internationaler Konsensusleitlinie bereits ergänzend die Zeit im Zielbereich („time in range“ [TIR]) als integraler Stoffwechselparameter herangezogen [20]. Sie ist definiert als der Prozentsatz an Zeit, in der sich der Glukosespiegel zwischen 70 mg/dl und 180 mg/dl bewegt, und sollte für alle Personen mit T1D über 70 % liegen. Seit dem Erscheinen dieser internationalen Konsensusleitlinie konnten diese Empfehlungen sehr rasch in die tägliche Praxis übernommen werden. Die Zeit im Zielbereich (sog. *grüner Bereich*) ist für PmD und deren Familien auch viel anschaulicher als der HbA_1c_-Wert. Es wird auch empfohlen, dass die Familien regelmäßig ihre Daten selbst auslesen und gemeinsam anschauen, was dann auch die Besprechung in der Diabetesambulanz vereinfacht.

### Sensorunterstützte Pumpentherapie, Low-Glucose-Suspension und Predictive-Low-Glucose-Suspension

Bei Kindern und Jugendlichen mit T1D werden Insulinpumpen häufig mit CGM-Systemen im Sinne einer sensorunterstützten Insulinpumpentherapie genutzt. Manche Hersteller liefern integrierte Systeme, bei denen die CGM-Werte nicht nur kontinuierlich auf dem Pumpendisplay angezeigt werden, sondern die Insulinabgabe automatisch, basierend auf aktuellen CGM-Werten, verändert wird. Als einfachste Variante gilt hier die automatisierte Unterbrechung der Insulinabgabe beim Erreichen niedriger Glukosewerte („low glucose insulin suspension“ [LGS]) bzw., wenn niedrige Glukosespiegel vorhergesagt werden („predictive low-glucose insulin suspension“ [PLGS]). Derartige Systeme der Firma Medtronic sind in Österreich bereits seit 2009 (LGS: Medtronic PARADIGM VEO) bzw. 2015 (PLGS oder „smartguard“: MiniMed 640G) auf dem Markt. Gemäß den Ergebnissen großer RCT (randomisierte kontrollierte Studie) zeigte sich ein Vorteil im Vergleich zur reinen sensorunterstützen Pumpentherapie bezüglich Reduktion der Häufigkeit und Dauer von, v. a. auch nächtlichen, Hypoglykämien [21–24]. Aufgrund höherer Glukosespiegelvariabilität bei gleichzeitig oft stark ausgeprägter Angst vor Hypoglykämien von Seiten der Eltern sind diese Systeme v. a. bei Kleinkindern und Kindern mit T1D sehr beliebt.

### Remote-Monitoring

Unterschiedliche CGM- oder AID-Systeme haben die zusätzliche Option eines Remote-Monitorings. Hierbei werden die Daten auf ein Handy übertragen und über Cloud-to-Cloud-Lösungen an sog. Follower. Im pädiatrischen Bereich sind das meist die Eltern, aber auch öfters Großeltern oder Betreuungseinrichtungen. Die Erwachsenen können damit die Glukosewerte ihrer Kinder überwachen, auch wenn diese in Bereuungseinrichtungen sind, und bei Bedarf bei Therapieentscheidungen unterstützen.

### Automatische Insulindosierung

Komplexere Ansätze, wie sie in sog. AID-Systemen (auch Closed-Loop-Systeme genannt [CL, sog. künstliche Bauchspeicheldrüse]) verfolgt werden, führen nicht nur zu einer Unterbrechung der vorprogrammierten Basalraten bei drohender Hypoglykämie, sondern zu einer kontinuierlichen Modulation der Insulinabgabe, abhängig von aktuellen und vergangenen CGM-Werten, über einen Algorithmus, der automatisch die optimale Menge an Insulin zur Vermeidung von Hypo- und Hyperglykämien berechnet. Die meisten aktuell in Verwendung und Testung befindlichen Systeme sind sog. Hybridsysteme, bei denen man zum Abdecken von Mahlzeiten weiterhin Blutzuckerwerte bzw. die Menge an Kohlenhydraten in die Pumpe eingeben muss.

Die Ergebnisse erster Studien zu AID-Systemen sind vielversprechend

**Figure 3:**
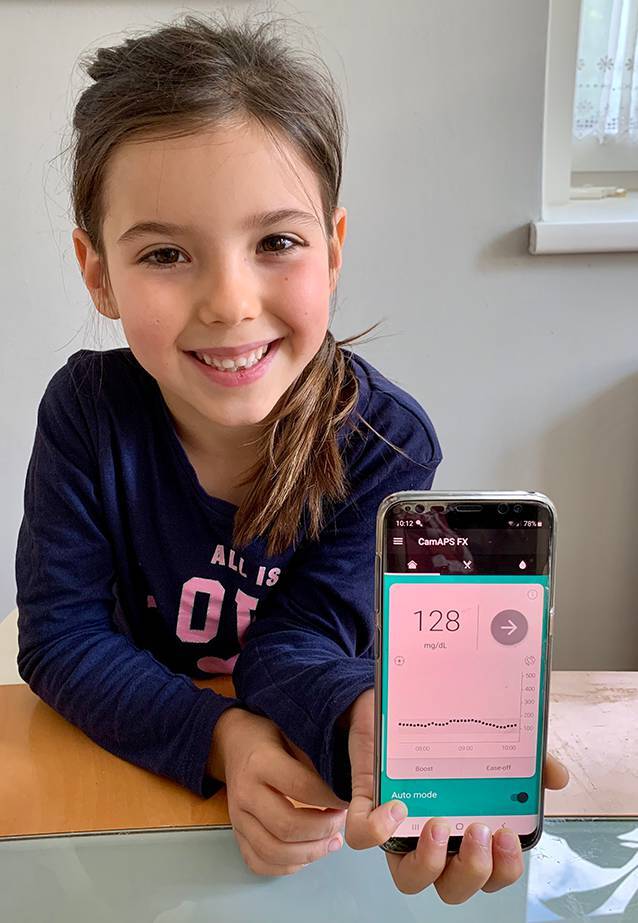

Resultate aus klinischen Studien von AID-Systemen sind vielversprechend im Hinblick auf eine Verbesserung der Stoffwechsellage bei gleichzeitiger Reduktion der Hypoglykämiehäufigkeit [25, 26]. Das erste dieser Systeme, Medtronic 670G, ist seit Juni 2019 auch in Österreich verfügbar und ab einem Alter von 7 Jahren zugelassen, seit Juli 2021 auch Medtronic 770G und seit Dezember 2021 Medtronic 780G, für deren Verwendung ist eine Mindesttagesinsulindosis von 8 IU/Tag erforderlich. Für die sehr junge Altersgruppe ab 1 Jahr ist das CamAPS-System zugelassen und seit Juli 2022 in Kombination mit der YpsoPump und dem DexCom G6 auch in Österreich verfügbar. In der multizentrischen, randomisierten Crossover-Studie KidsAP (Horizon 2020-EU-Projekt [EU: Europäische Union], mit einer österreichischen Beteiligung) wurden mit CamAPS im Vergleich zu einer sensorunterstützen Pumpentherapie bei sehr jungen Kindern (1–7 Jahre) eine signifikante Verbesserung der TIR und eine Reduktion der Hyperglykämien ohne Erhöhung der Hypoglykämien beobachten ([27]; Abb. 3). In weiteren Publikationen dieser KidsAP-Studie wurde auch über eine Verbesserung der Lebensqualität der Familien, weniger Stress und besseren Schlaf berichtet [28, 29].

Weitere AID-Systeme (u. a. Tandem Control-IQ, Omnipod 5 FX, Diabeloop), die bereits über eine CE-Zertifizierung verfügen, sind in weiten Teilen Europas – so auch in Österreich – noch nicht auf dem Markt. Im September 2022 wurde zu AID-Systemen eine internationale Konsensusempfehlung publiziert [37]. Eine Übersicht der Systeme mit CE-Zertifizierung und deren Unterschiede sind in Tab. 1 aufgelistet.

**Table 1:** 

	CamAPS FX	Diabeloop DBLG1/DBL4T	Medtronic 670G	Medtronic 770G	Medtronic 780G	Omnipod 5	t:slim X2 mit Control-IQ
Insulinpumpe	DANA R/S, DANA‑i oder mylife YpsoPump	Accu-Chek Insight^a^	MiniMed 670G	MiniMed 770G	MiniMed 780G	Omnipod 5 ACE	t:slim X2
		Kaleido-Patch-Pumpe					
Glukosesensor	DexCom G6	DexCom G6	Guardian 3	Guardian 3	Guardian 4	DexCom G6	DexCom G6
Funktionsdauer des Sensors	10 Tage	10 Tage	7 Tage	7 Tage	7 Tage	7 Tage	10 Tage
Nötige Blutzuckerspiegelkontrollen	Keine	Keine	Mindestens 2‑ bis 3‑mal/Tag	Mindestens 2‑ bis 3‑mal/Tag	Keine	Keine	Keine
Art des Algorithmus	MPC	MPC	PID	PID	PID mit Fuzzylogik und MPC-Anteil	MPC	MPC
Plattform des Algorithmus	Android-Smartphone (iOS geplant)	Handgerät	In der Pumpe	In der Pumpe	In der Pumpe	Handgerät	In der Pumpe
Altersbeschränkung	> 1 Jahr	12–18 Jahre (DBL4T)	> 7 Jahre	> 7 Jahre	> 7 Jahre	> 2 Jahre	> 6 Jahre
	(auch für Schwangere)	> 18 Jahre (DBLG1)					
Glukoseziel (mg/dl)	80–200	100–180	120	120	100, 110 oder 120	110–150	110
Automatische Korrekturbolusgabe	Nein	Ja	Nein	Nein	Ja	Nein	Ja
Möglichkeit der Datenverfügbarkeit	Automatisch, diasend	Download, diasend (Sensordaten CLARITY)	Download, CareLink	Automatisch, CareLink	Automatisch, CareLink	Automatisch, Omnipod Connect	Download, diasend/Glooko (Sensordaten CLARITY)
Sonstiges	Boost-Modus	Variable Aggressivität	–	Per Handy anzusehen	Per Handy anzusehen	Schlauchlos	Nachtmodus
Temporäres Ziel erhöhen	„Ease off“/Aktivitätsmodus	Zen-Modus (20–40 mg/dl höher als aktuelles Ziel)	Temporäres Ziel (150 mg/dl)	Temporäres Ziel (150 mg/dl)	Temporäres Ziel (150 mg/dl)	Aktivitätsmodus (150 mg/dl)	Aktivitätsmodus
Verfügbar in Österreich (09/2022)	Ja (mit mylife YpsoPump)	Nein	Ja	Ja^b^	Ja	Nein	Nein

*AID* automatische Insulindosierung, *MPC* „model predictive control“, *PID* „proportional integral derivative“

^a^Accu-Chek Insight wird noch 2022 vom Markt genommen

^b^Wurde von Juli bis November 2021 in Österreich angeboten, wurde von 780G abgelöst

Bihormonale Systeme, die u. a. zusätzlich zu Insulin auch Glukagon zur Vermeidung von Hypoglykämien abgeben, befinden sich in klinischer Testung.

Getriggert durch die immer noch begrenzte Verfügbarkeit von AID-Systemen, deren Entwicklung und Zulassung als Medizinprodukt streng behördlich geregelt ist und gewisse Zeit in Anspruch nimmt, bildete sich in den letzten Jahren eine immer größer werdende Community, die gemäß ihrem Mantra (#wearenotwaiting) die Entwicklung und Verbreitung von DIY-Open-Source-AID-Systemen (DIY: „do it yourself“, z. B. OpenAPS, AndroidAPS oder LOOP) vorantreibt [30]. Immer mehr Menschen mit T1D fertigen sich so ihre AID-Systeme selbst an, über online frei verfügbare Anleitungen und Algorithmen, die von der Community auch ständig modifiziert werden können und sich im eigenverantwortlichen Selbstexperiment durch den/die User:in der behördlichen Zulassung entziehen. Im Jahr 2022 wurde zu diesen Open-Source-AID-Systemen erstmalig auch eine Konsensusempfehlung veröffentlicht, die als Hilfe für medizinisches Fachpersonal dienen soll [31].

## Technologie als Grundlage für telemedizinische Betreuung

Alle Insulinpumpen, CGM- und AID-Systeme können über cloudbasierte Software ausgelesen werden bzw. werden automatisch in die entsprechende Cloud hochgeladen. Damit besteht die ideale technische Grundlage für eine telemedizinische Betreuung, die sich v. a. während der SARS-CoV‑2 Pandemie (SARS-CoV-2: „severe acute respiratory syndrome coronavirus 2“) in vielen Diabeteszentren zu einer neuen Realität entwickelte [32, 33]. Auch über die Pandemie hinaus birgt die Telemedizin großes Potenzial in der Langzeitbetreuung von Menschen mit Diabetes mellitus. Um sie in die Versorgungsstruktur implementieren zu können, bedarf es allerdings einer soliden Planung und Umsetzung unter Berücksichtigung rechtlicher und datenschutzrechtlicher Grundlagen.

## Vermittlung von Theorie und Praxis der Diabetestechnologie

Die Implementierung und Verwendung von Diabetestechnologie müssen fundiert und altersgerecht vermittelt und trainiert werden [33]. Strukturierte formale Schulungsprogramme erwiesen sich im Sinne von verbesserter glykämischer Kontrolle, Akzeptanz und Zufriedenheit der Anwender:innen als effektiv [34–36]. Gerade bei jungen Kindern ist es essenziell, auch alle Betreuungspersonen des Kindes zu schulen. Das multidisziplinäre Schulungsteam sollte (pädiatrische) Diabetolog:innen, Diabetesberater:innen, Diätolog:innen, Psycholog:innen sowie Sozialarbeiter:innen umfassen.

Das multidisziplinäre Team muss die Datenanalyse beherrschen, um entsprechende Therapieanpassungen vornehmen zu können. Um eine qualitativ hochwertige Versorgung zu gewährleisten, sind kontinuierliche Fortbildungen der Diabetesteams unverzichtbarer Teil eines erfolgreichen Qualitätsmanagements.

## Fazit für die Praxis

- Innovationen im Bereich der Diabetestechnologie verbesserten und erleichterten die Therapie und das Management für Kinder- und Jugendliche mit Typ‑1-Diabetes und ihre Familien.
- Ein bemerkenswerter Meilenstein wurde zuletzt mit der Translation von Forschung in die klinische Anwendung algorithmisch gesteuerter AID-Systeme (AID: automatische Insulindosierung) erreicht.
- Diese sind in ihrer Verwendung auch in der pädiatrischen Altersgruppe sicher, verbessern die TIR („time in range“ [Zeit im Zielbereich]) und reduzieren die psychosozialen Belastungen des Alltags.
- Die Herausforderung in der Betreuung ist es, aus der immer größer werdenden Auswahl an technischen Lösungen das für die Betroffenen und deren Familien im entsprechenden Lebensabschnitt bestgeeignetste System zu finden.
- Weitere Forschung und Post-Marketing-Analysen von Diabetestechnologien in allen Altersstufen sind essenziell, um entsprechende Evidenz für diese Entscheidung zu schaffen.
